# Supplementary material for: Resolving Entangled JH-H-Coupling Patterns for Steroidal Structure Determinations by NMR Spectroscopy
Source: Molecules. 2021 Apr 30;26(9):2643. doi: 10.3390/molecules26092643 (PMC8124291; doi:10.3390/molecules26092643)
Supplement: Supplementary file 1 [file molecules-26-02643-s001.zip › molecules-1193038-supplementary.pdf]

Resolving Entangled $J_{H\text{-}H}$-Coupling Patterns for Steroidal Structure Determinations by NMR Spectroscopy

Danni Wu [1], Kathleen D. Carillo [1,2,3], Jiun-Jie Shie [1], Steve S.-F. Yu [1] and Der-Lii M. Tzou [1,4,*]

[1] Institute of Chemistry, Academia Sinica, Nankang, Taipei 11529, Taiwan ROC

[2] Taiwan International Graduate Program, Sustainable Chemical Science and Technology (SCST), Academia Sinica, Nankang, Taipei 11529, Taiwan

[3] The Department of Applied Chemistry, National Yang Ming Chiao Tung University, Hsinchu 30013, Taiwan

[4] Department of Applied Chemistry, National Chia-Yi University, Chia-Yi 60004, Taiwan, ROC

Supplementary information

*To whom correspondence should be addressed: Dr. Der-Lii M. Tzou, Institute of Chemistry, Academia Sinica 128, Academia Rd., Sec. 2, Nankang, Taipei 11529, Taiwan, Republic of China, Tel: +886 2 55728524, fax: +886 2 55721237, E-mail: tzougate@gate.sinica.edu.tw

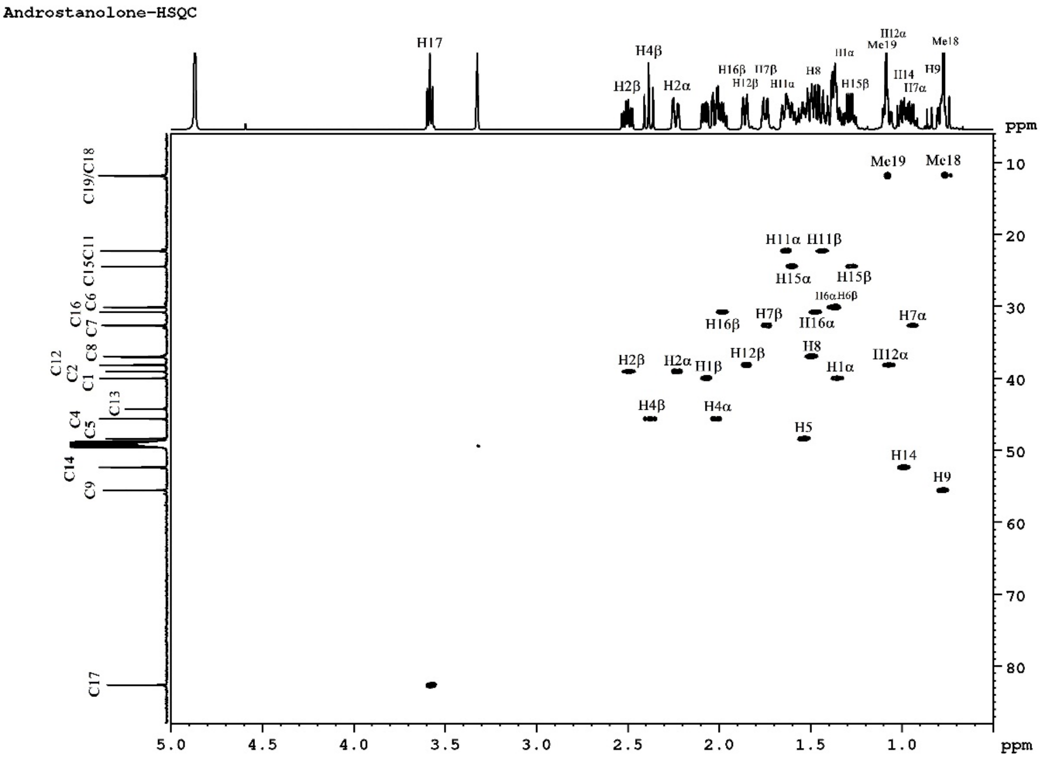

Figure S1. Solution $^{1}H/^{13}C$ 2D HSQC NMR spectrum of **1** dissolved in *d4*-MeOH solution. The ^{1}H and ^{13}C chemical shift assignments were indicated on each of the projection profiles. All ^{1}H and ^{13}C chemical shifts are tabulated in Tables 1 & S1.

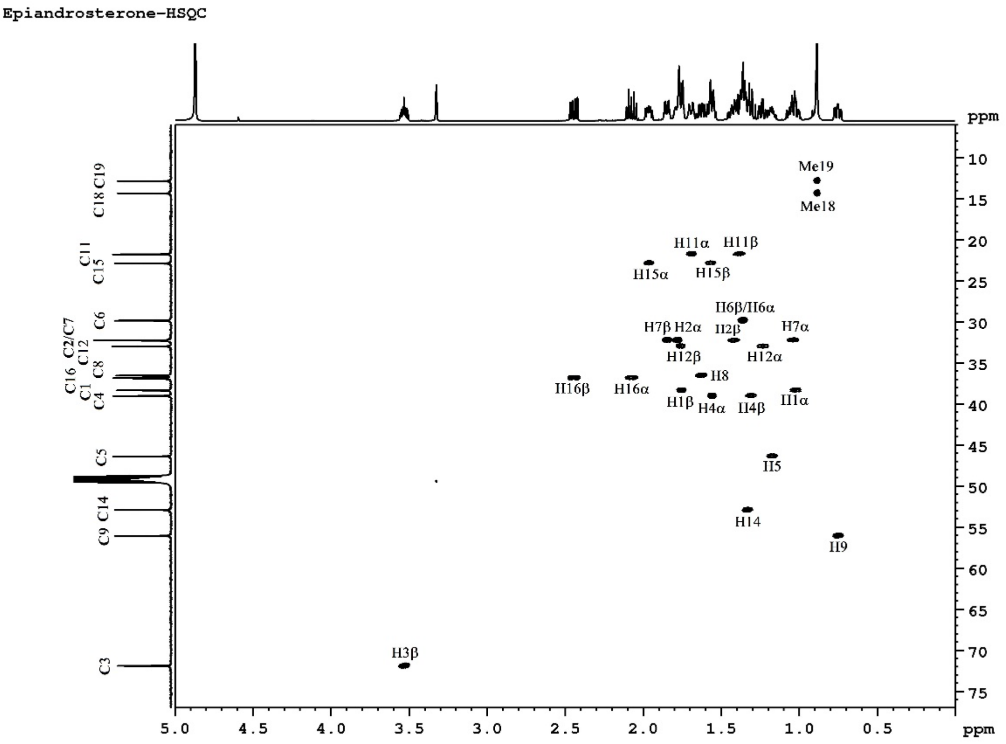

Figure S2. Solution $^{1}H/^{13}C$ 2D HSQC NMR spectrum of **2** dissolved in *d4*-MeOH solution. The ^{1}H and ^{13}C chemical shift assignments were indicated on each of the projection profiles. All ^{1}H and ^{13}C chemical shifts are tabulated in Tables 1 & S1.

(a)

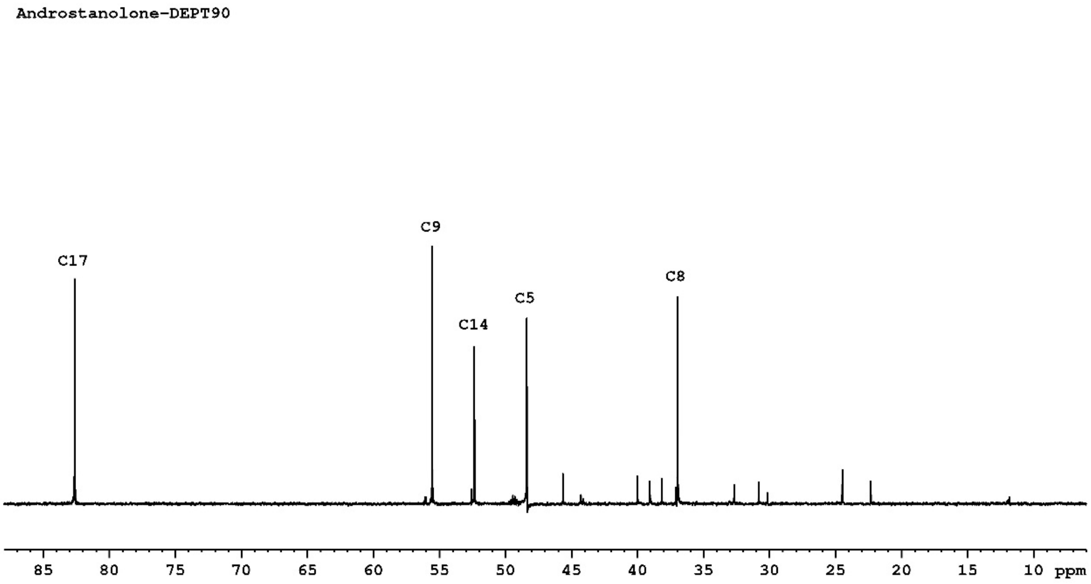

(b)

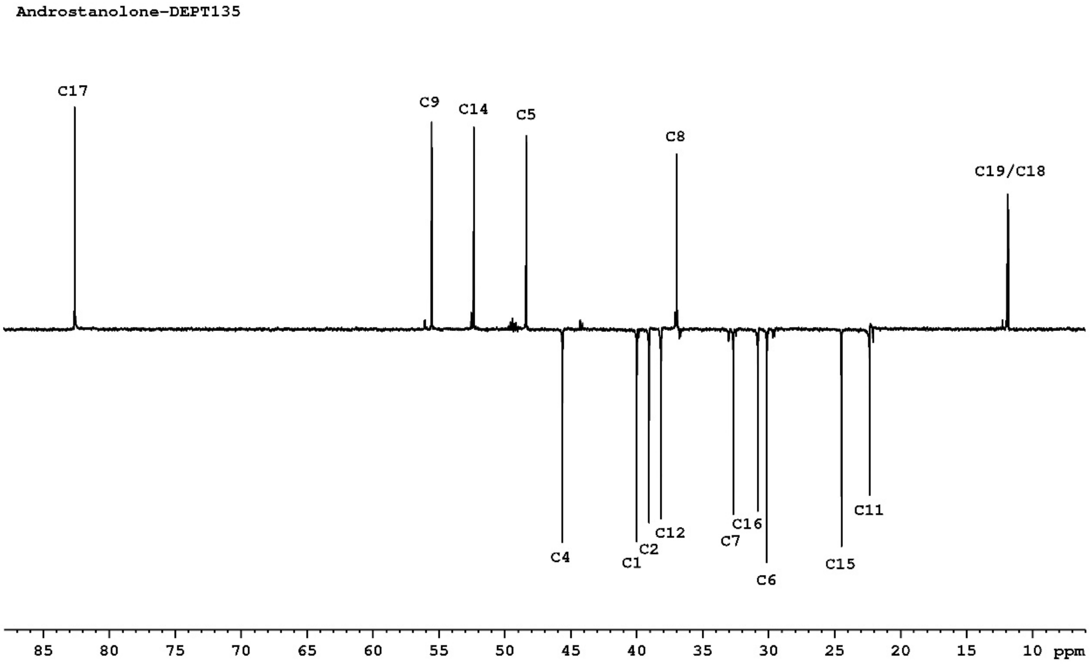

Figure S3. Solution $^{1}H/^{13}C$ DEPT NMR spectrum of **1** dissolved in *d4*-MeOH solution. (a) Solution DEPT-90 spectrum and (b) DEPT-135 spectrum of **1**. The ^{13}C chemical shift assignments were indicated on each of the ^{1}H correlated ^{13}C signals. All ^{1}H and ^{13}C chemical shifts are tabulated in Tables 1 & S1.

(a)

(b)

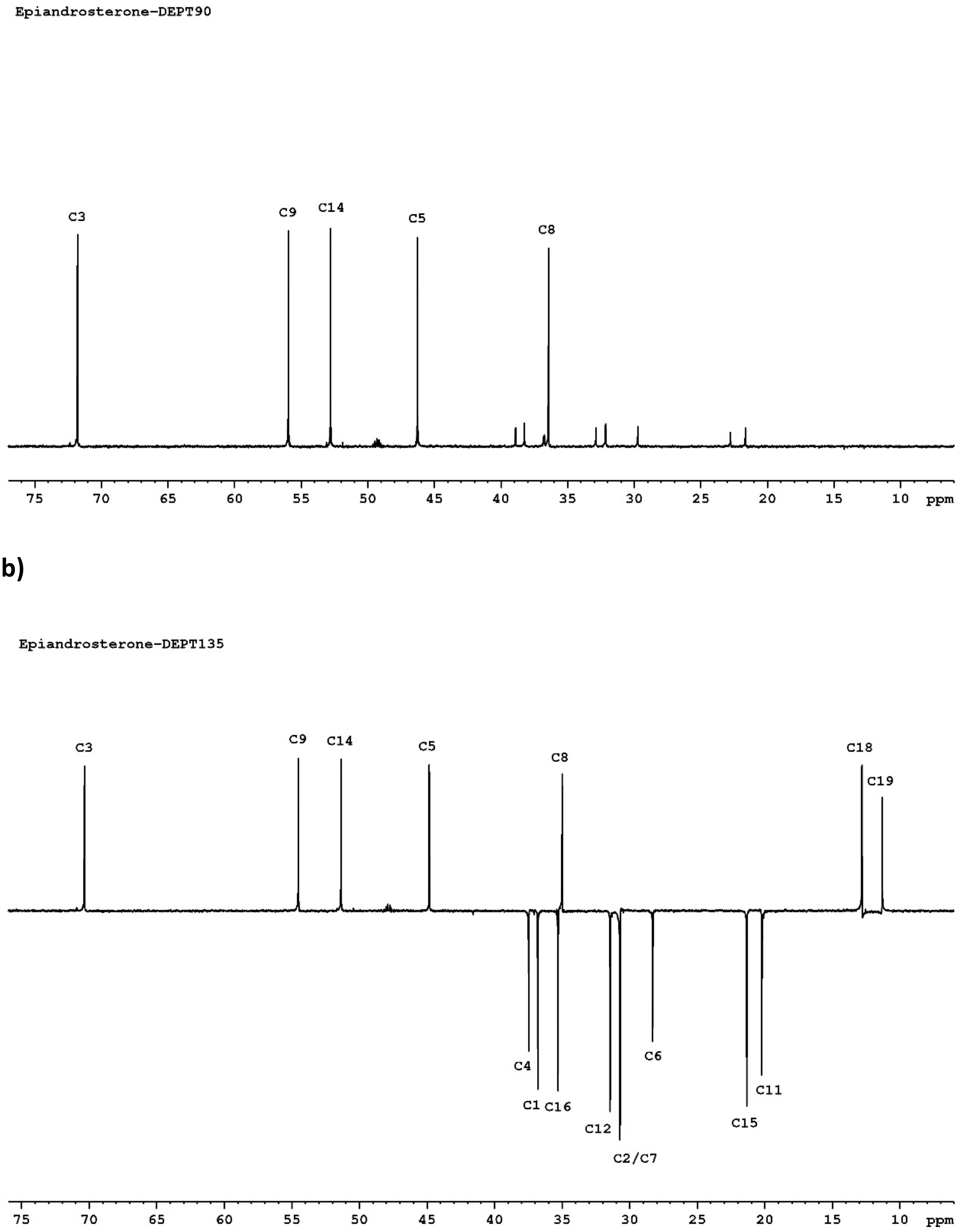

Figure S4. Solution $^{1}H/^{13}C$ DEPT NMR spectrum of **2** dissolved in *d4*-MeOH solution. (a) Solution DEPT-90 spectrum (b) and DEPT-135 spectrum of **2**. The ^{13}C chemical shift assignments were indicated on each of the ^{1}H correlated ^{13}C signals. All ^{1}H and ^{13}C chemical shifts are tabulated in Tables 1 & S1.

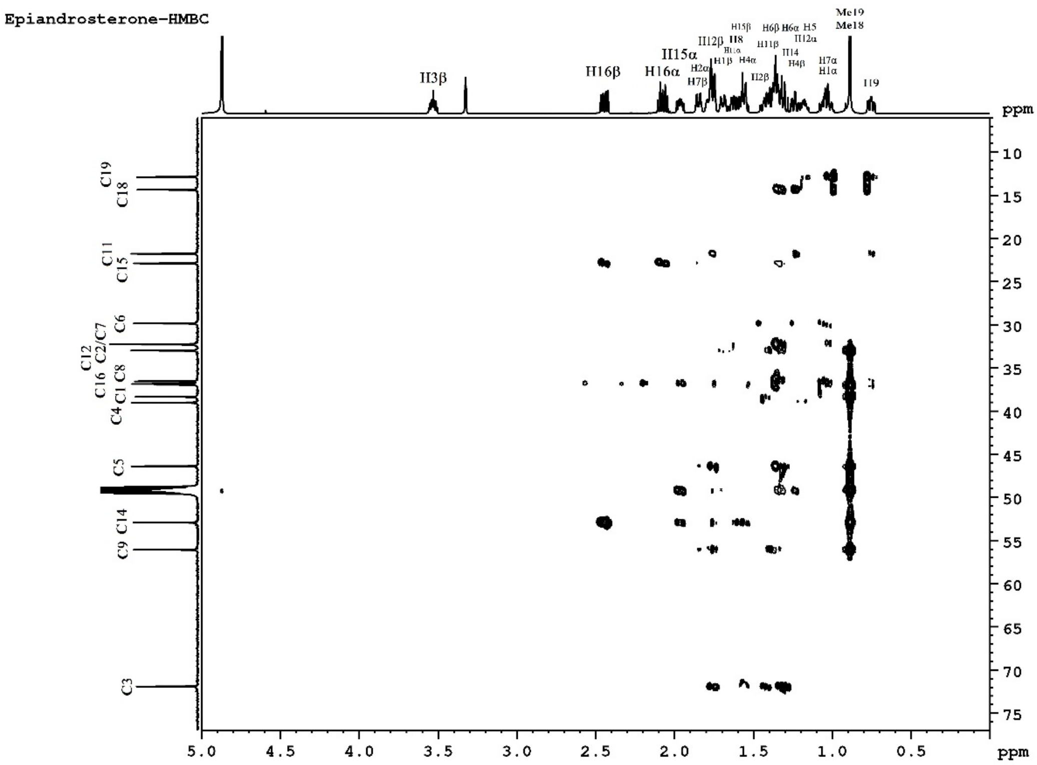

Figure S5. Solution $^{1}H/^{13}C$ 2D HMBC NMR spectrum of **1** dissolved in *d4*-MeOH solution. The ^{1}H and ^{13}C chemical shift assignments were indicated on each of the projection profiles. All ^{1}H and ^{13}C chemical shifts are tabulated in Tables 1 & S1.

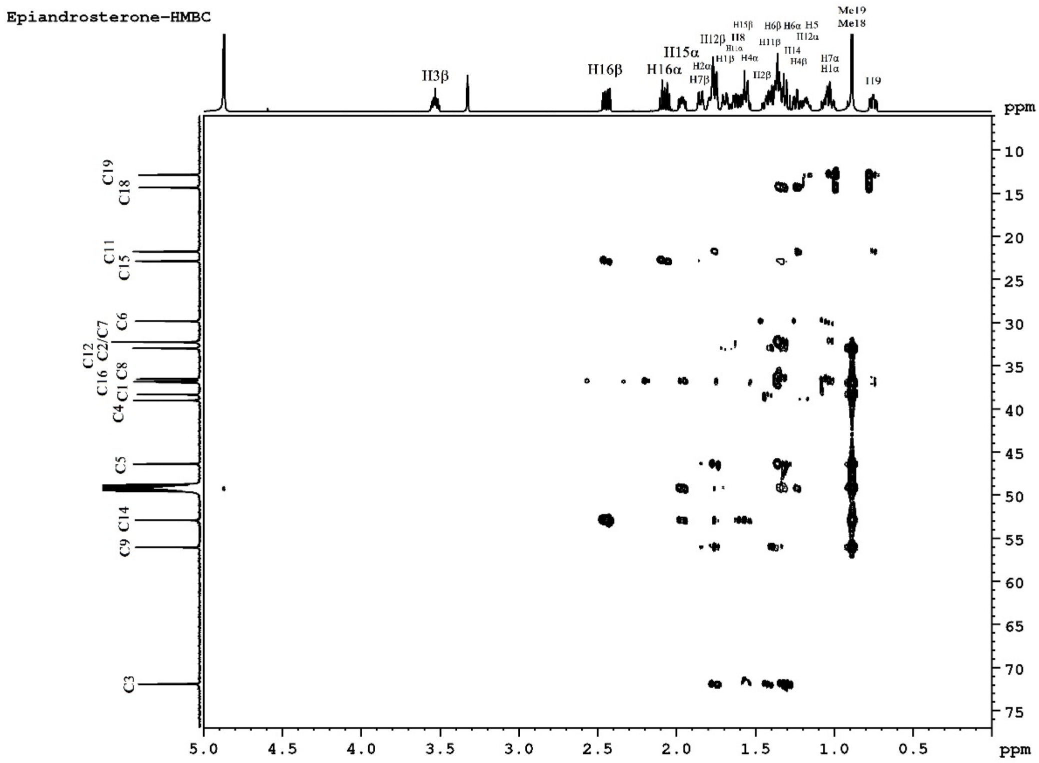

Figure S6. Solution $^{1}H/^{13}C$ 2D HMBC NMR spectrum of **2** dissolved in *d4*-MeOH solution. The ^{1}H and ^{13}C chemical shift assignments were indicated on each of the projection profiles. All ^{1}H and ^{13}C chemical shifts are tabulated in Tables 1 & S1.

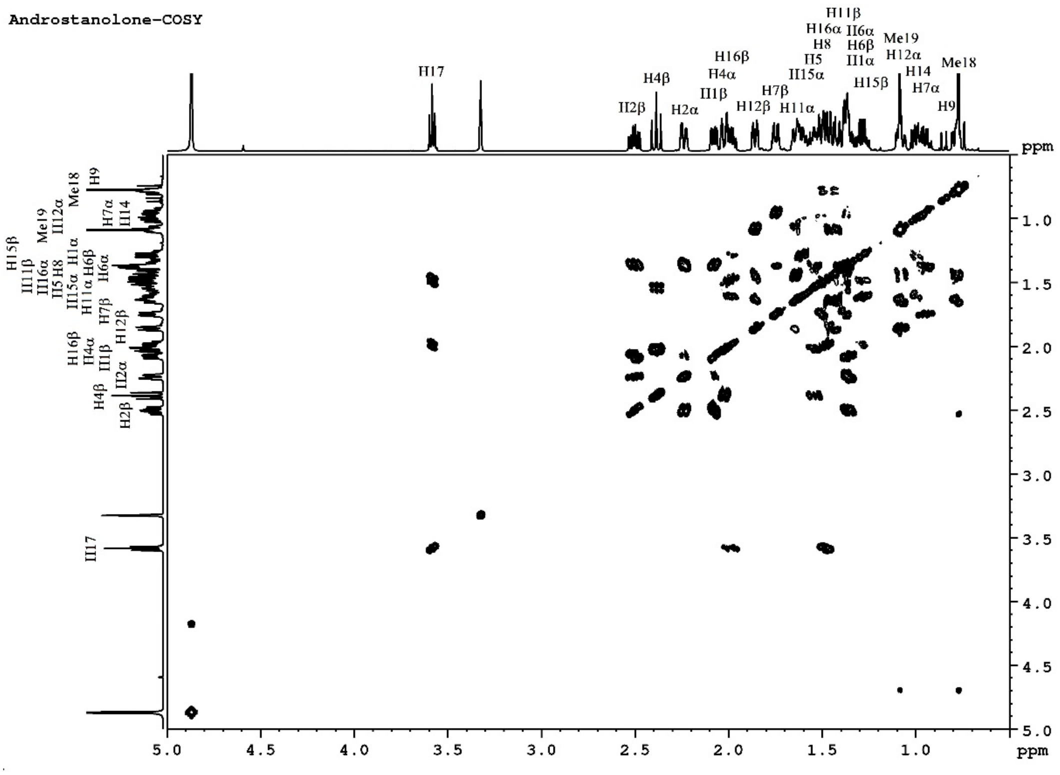

Figure S7. Solution 2D COSY NMR spectrum of **1** dissolved in *d4*-MeOH solution. The ^{1}H chemical shift assignments were indicated on each of the projection profiles. All ^{1}H chemical shifts are tabulated in Table 1.

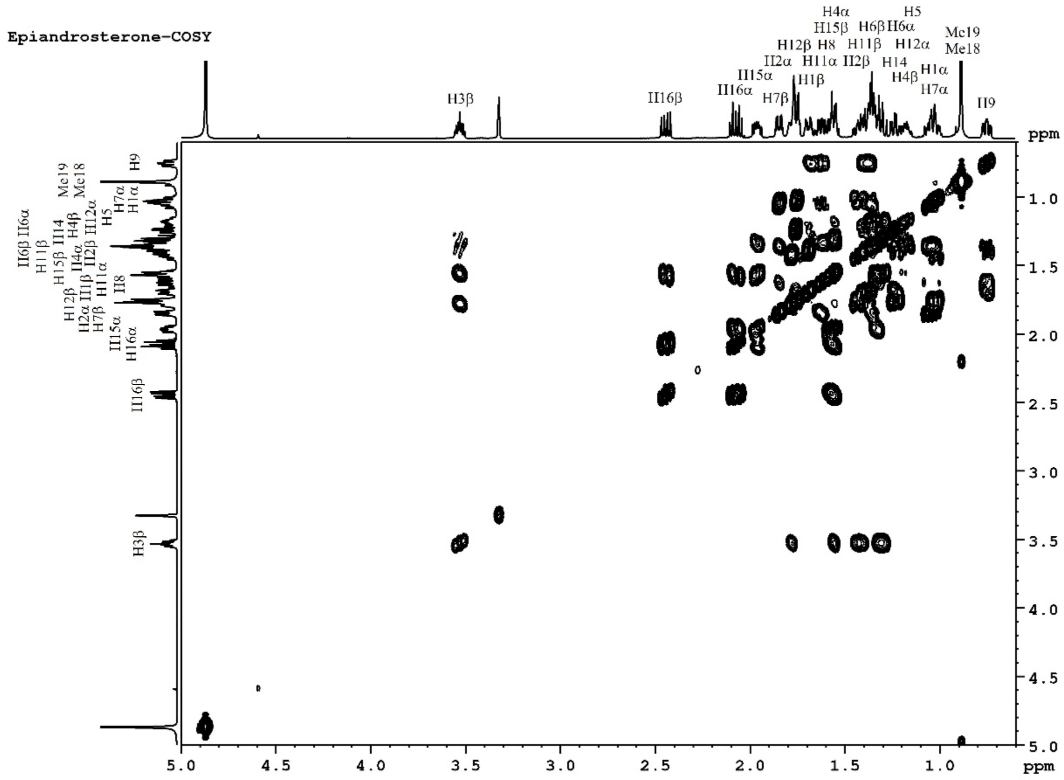

Figure S8. Solution 2D COSY NMR spectrum of **2** dissolved in *d4*-MeOH solution. The ^{1}H chemical shift assignments were indicated on each of the projection profiles. All ^{1}H chemical shifts are tabulated in Table 2.

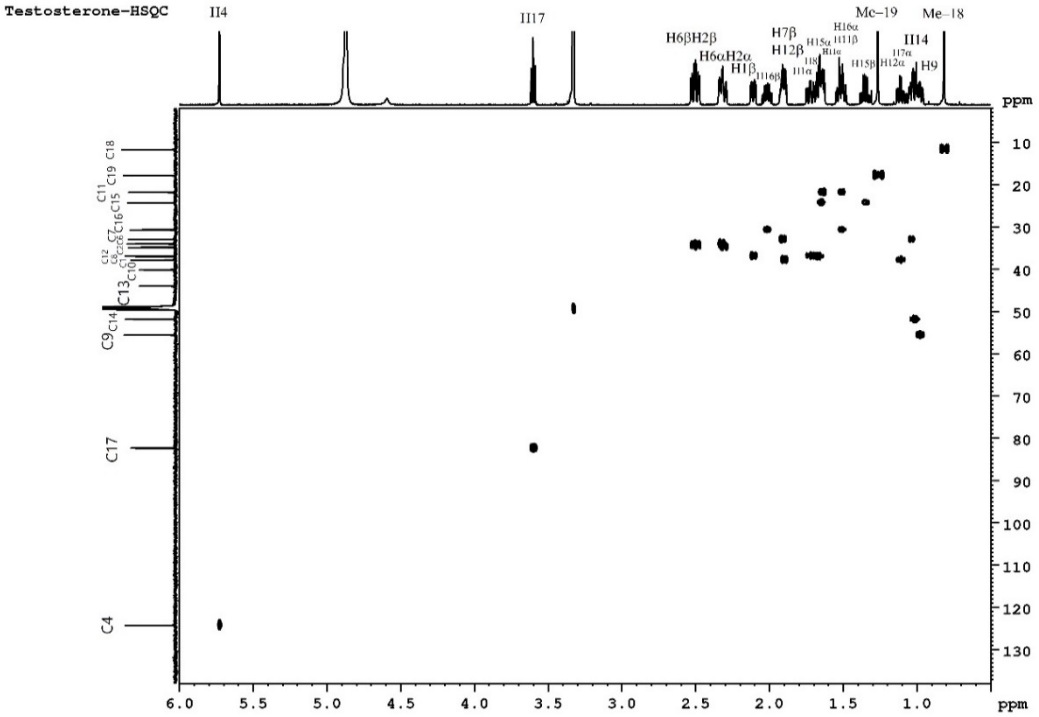

Figure S9. Solution $^{1}H/^{13}C$ 2D HSQC NMR spectrum of **3** dissolved in *d4*-MeOH solution. The ^{1}H and ^{13}C chemical shift assignments were indicated on each of the projection profiles.

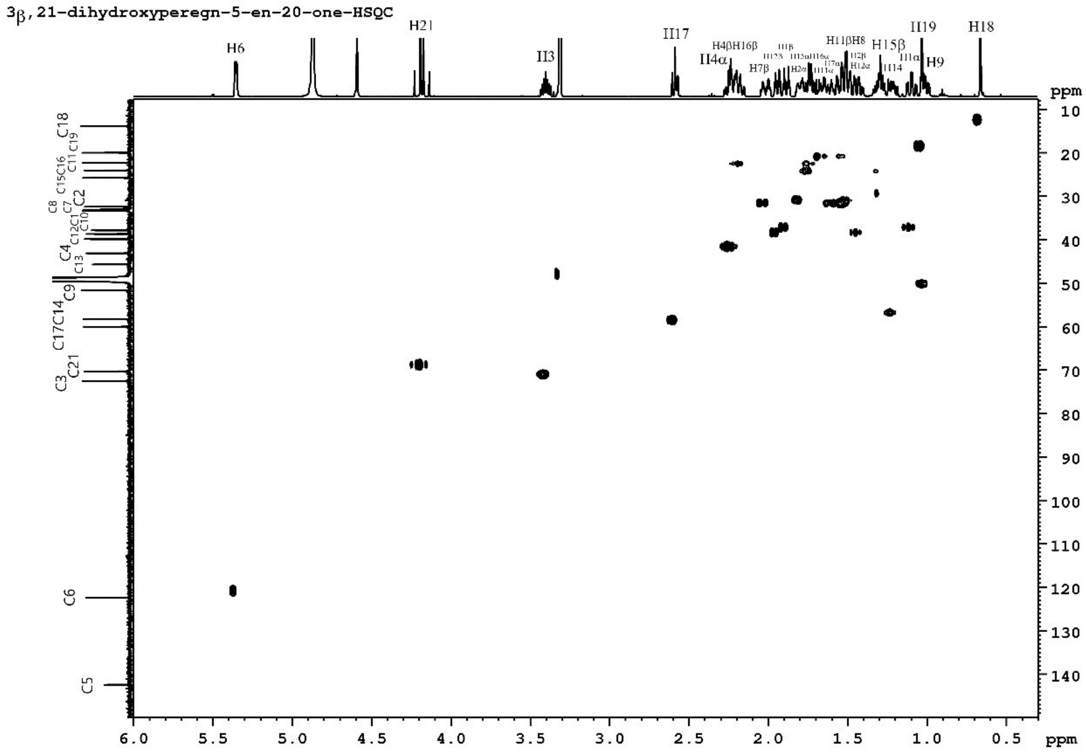

Figure S10. Solution $^1H/^{13}C$ 2D HSQC NMR spectrum of **4** dissolved in *d4*-MeOH solution. The ^1H and ^{13}C chemical shift assignments were indicated on each of the projection profiles.

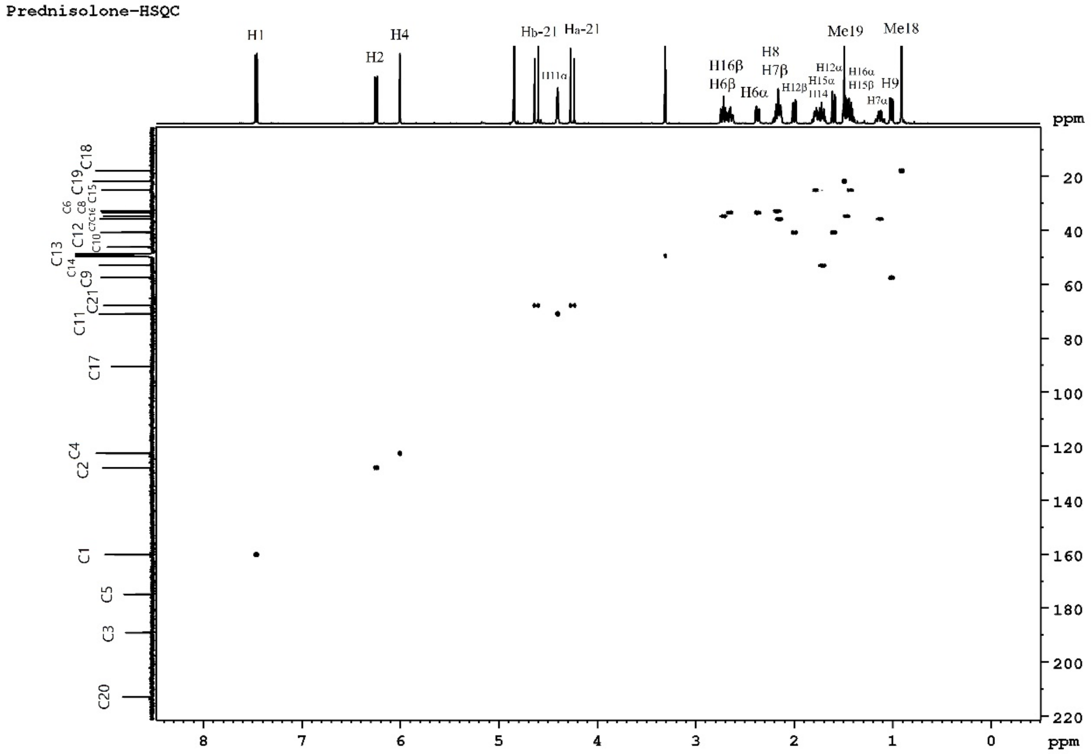

Figure S11. Solution $^{1}H/^{13}C$ 2D HSQC NMR spectrum of **5** dissolved in *d4*-MeOH solution. The ^{1}H and ^{13}C chemical shift assignments were indicated on each of the projection profiles.

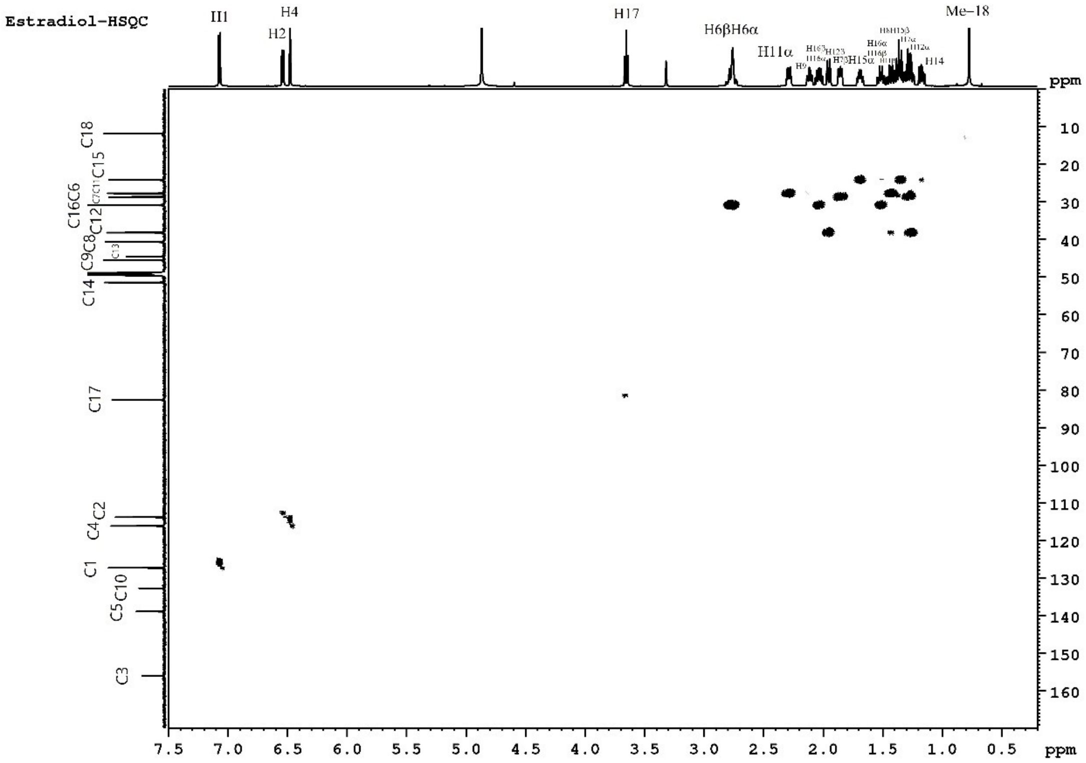

Figure S12. Solution $^{1}H/^{13}C$ 2D HSQC NMR spectrum of **6** dissolved in *d4*-MeOH solution. The ^{1}H and ^{13}C chemical shift assignments were indicated on each of the projection profiles.

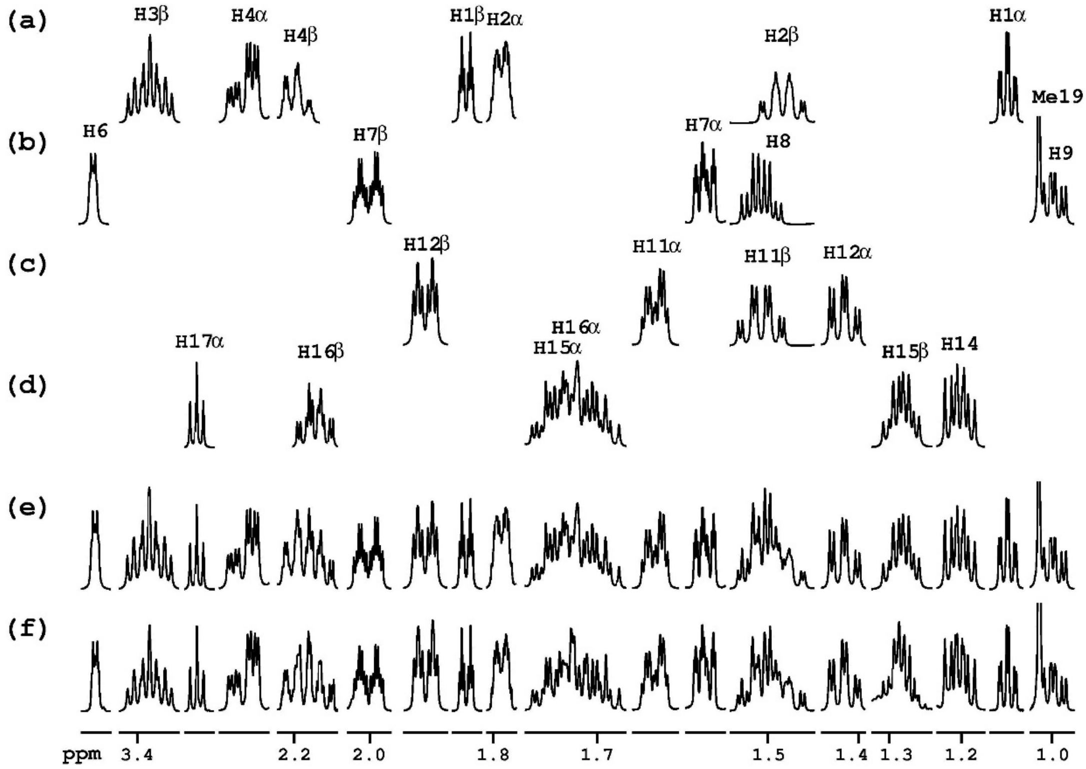

Figure S13. Characteristic ^{1}H NMR segmental spectra patterns of steroidal compound **4**. Four subspectra were generated using the Daisy software program, corresponding to steroidal rings (a) *A*, (b) *B*, (c) *C* and (d) *D*, respectively. Given the ^{1}H chemical shifts deduced from the 2D HSQC measurements (Supporting Information Fig. S10) and associated J_{H-H} coupling constants determined from **1** and **2** (Table 1), one was able to construct the ^{1}H NMR subspectra and to identify spectral patterns in different steroidal compounds; for details see text. (e) A summation of the four subspectra (a), (b), (c) and (d) in the simulation of the respective ^{1}H NMR spectrum. (f) Experimental ^{1}H NMR spectrum. For better clarity, the subspectra are presented on different scales.

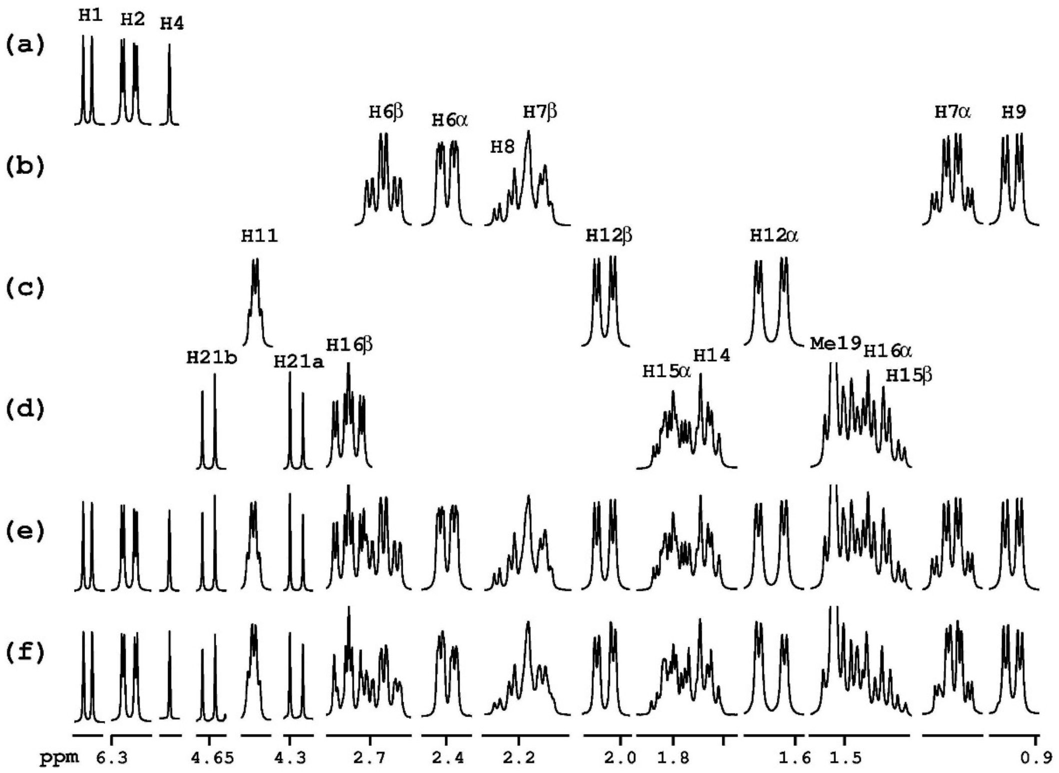

Figure S14. Characteristic ^{1}H NMR segmental spectra patterns of steroidal compound **5**. Four subspectra were generated using the Daisy software program, corresponding to steroidal rings (a) *A*, (b) *B*, (c) *C* and (d) *D*, respectively. Given the ^{1}H chemical shifts deduced from the 2D HSQC measurements (Supporting Information Fig. S11) and associated $J_{H\text{–}H}$ coupling constants determined from **1** and **2** (Table 1), one was able to construct the ^{1}H NMR subspectra and to identify spectral patterns in different steroidal compounds; for details see text. (e) A summation of the four subspectra (a), (b), (c) and (d) in the simulation of the respective ^{1}H NMR spectrum. (f) Experimental ^{1}H NMR spectrum. For better clarity, the subspectra are presented on different scales.

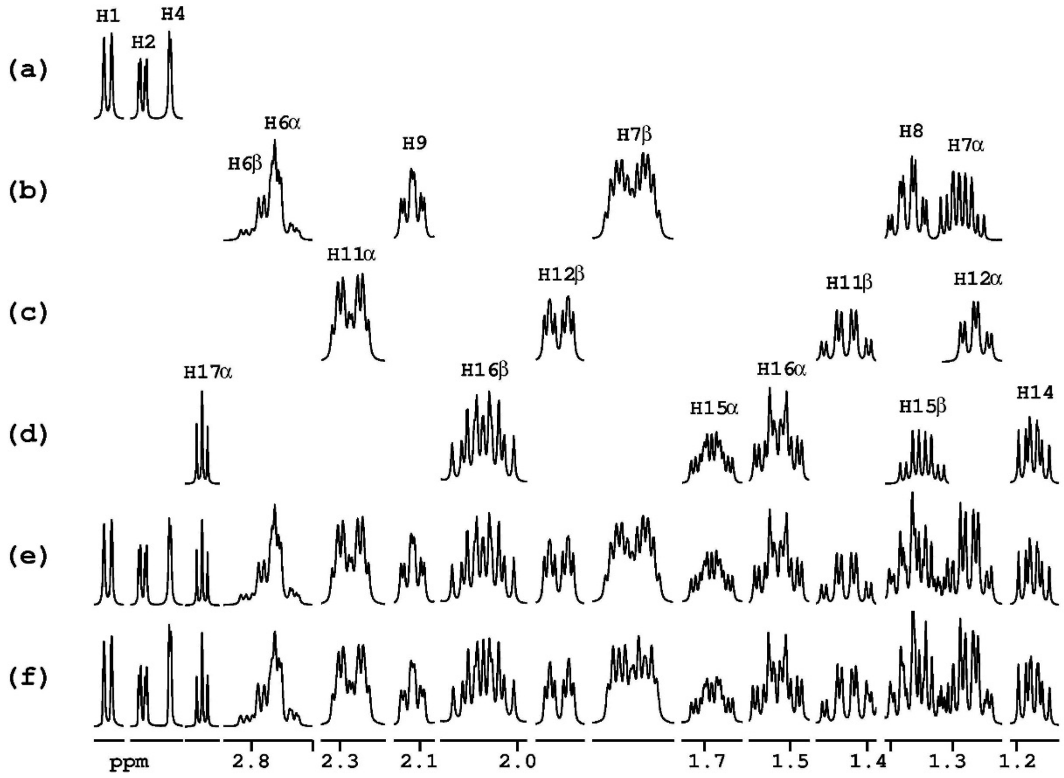

Figure S15. Characteristic ^{1}H NMR segmental spectra patterns of steroidal compound **6**. Four subspectra were generated using the Daisy software program, corresponding to steroidal rings (a) *A*, (b) *B*, (c) *C* and (d) *D*, respectively. Given the ^{1}H chemical shifts deduced from the 2D HSQC measurements (Supporting Information Fig. S12) and associated $J_{H\text{–}H}$ coupling constants determined from **1** and **2** (Table 1), one was able to construct the ^{1}H NMR subspectra and to associate the spectral similarities among different steroidal compounds; for details see text. (e) A summation of the four subspectra (a), (b), (c) and (d) in the simulation of the respective ^{1}H NMR spectrum. (f) Experimental ^{1}H NMR spectrum. For better clarity, the subspectra are presented on different scales.

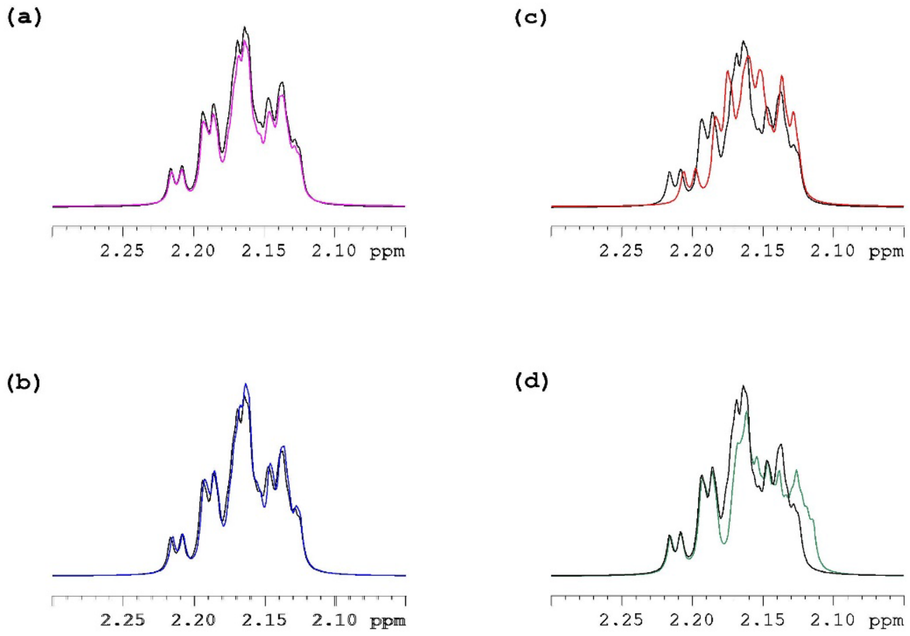

Figure S16. Superimpositions of multiple doublet splitting patterns of overlapped signals of **5** in various combinations. Simulations of two overlapped H7β and H8 signal patterns at 2.137 ppm (12.86, 5.6, 4.1, 1.87 Hz) & 2.177 ppm (12.32, 11.25, 10.9, 4.1 Hz) colored in black. (a) simulation colored in pink; H7β same as black, and H8: 2.177 ppm (12.32, 11.25, 10.9, 3.8 Hz), with a coupling constant deviation 0.3 Hz; (b) simulation colored in blue; H7β same as black, and H8: 2.167 ppm (12.32, 11.25, 10.9, 4.1 Hz), with a deviation 0.01 ppm in the chemical shifts, (c) simulation colored in red; H8 same as black, and H7β: 2.137 ppm (12.86, 5.6, 4.1, 2.17 Hz), with a coupling constant deviation 0.3 Hz; (d) simulation colored in green; H8 same as black, and H7β: 2.127 ppm (12.86, 5.6, 4.1, 1.87 Hz), with a deviation 0.01 ppm in the chemical shifts. As suggested by the spectral differences, one is able to refine the associated $J_{H\text{-}H}$ values and their chemical shifts from for the curve fitting, revealing an accuracy of 0.01 ppm for the chemical shifts and 0.3 Hz for the $J_{H\text{-}H}$ values. A linewidth of 1.5 Hz was added into these simulations to mimic the line broadening of experimental data in the curve fitting simulation process.

Table S1. ^{13}C chemical shift assignments of Androstanolone (**1**) and Epiandrosterone (**2**) [a]

Carbon	^{13}C chemical shifts (ppm)	
	**1**	**2**
C1	40.0	38.3
C2	39.1	32.3
C3	214.9	71.9
C4	45.6	39.0
C5	48.4	46.4
C6	30.1	29.8
C7	32.7	32.2
C8	37.0	36.5
C9	55.5	56.1
C10	37.1	36.9
C11	22.3	21.8
C12	38.1	33.0
C13	44.3	49.3
C14	52.4	52.9
C15	24.5	22.9
C16	30.8	36.8
C17	82.6	224.2
C18	11.8	14.3
C19	11.9	12.8

[a] Chemical shift are in units of ppm referenced to the Methanol-d_4 at 49.15 ppm, within an uncertainty of ± 0.1 ppm and was examined by AV500.

Table S2. H(X)-C-C-H(Y) dihedral angles deduced from the vicinal $^3J_{H-H}$ coupling constants of **1**

X-Y	NMR [a]	X-ray [b]	Ab initio
1α-2α	49.9	47.0	52.8
1β-2α	72.5	67.7	68.8
1β-2β	42.5	51.4	42.6
4α-5	60.0	52.0	59.5
5-6α	60.0	58.0	60.7
5-6β	159.4	179.4	160.1
6α-7α	49.9	60.2	52.1
6α-7β	68.3	55.5	65.9
6β-7α	162.0	174.5	166.5
6β-7β	63.9	58.7	48.4
7α-8	162.0	173.1	174.7
7β-8	58.6	54.1	56.4
8-9	154.2	172.7	155.8
8-14	152.8	173.3	156.4
9-11α	60.0	71.9	62.0
9-11β	168.8	165.0	169.4
11α-12α	60.0	63.9	64.4
11α-12β	68.3	68.7	68.4
11β-12β	58.6	39.7	38.3
14α-15α	36.2	40.7	39.8
14α-15β	160.3	164.0	164.1
15α-16α	20.8	9.2	18.0
15α-16β	111.4	112.8	112.2
15β-16α	125.8	119.0	138.9
16α-17α	31.6	30.1	29.6
16β-17α	142.0	137.9	144.6

[a] The dihedral angles in degree deduced from the vicinal $^3J_{H-H}$ coupling constants according to Bothner-By equation [20].

[b] The dihedral angles extracted from the X-ray structure of **1** reported in literature [21].

Table S3. H(X)-C-C-H(Y) dihedral angles deduced from the vicinal $^3J_{H-H}$ coupling constants of **2**

X-Y	NMR [a]	X-ray [b]	Ab initio
1α-2α	56.5	62.5	55.9
1β-2α	72.5	54.7	69.0
1β-2β	63.9	68.4	60.9
2α-3	57.2	50.9	53.3
2β-3	158.6	169.6	175.4
3-4α	53.1	58.7	51.3
3-4β	153.5	177.3	156.6
4α-5	63.9	57.7	60.4
4β-5	167.4	173.8	170.0
5-6α	60.0	57.6	56.2
5-6β	162.0	173.8	163.5
6α-7α	60.0	53.0	62.8
6α-7β	68.3	63.6	62.1
6β-7α	180.0	170.3	169.1
6β-7β	63.9	53.8	66.0
7α-8	162.0	178.6	162.7
7β-8	58.6	53.7	41.2
8-9	154.2	178.9	158.1
8-14	152.8	178.5	152.3
9-11α	60.0	58.9	62.5
9-11β	168.8	174.6	169.6
11α-12α	60.0	60.0	61.3
11α-12β	68.3	58.2	65.0
11β-12β	58.6	54.6	41.6
14α-15α	48.1	47.0	48.6
14α-15β	168.8	167.0	169.6
15α-16α	30.2	22.2	27.6
15β-16α	143.1	143.5	155.2

15β-16β	27.4	13.1	23.8

[a] The dihedral angles in degree deduced from the vicinal $^{3}J_{H-H}$ coupling constants according to Bothner-By equation [20].

[b] The dihedral angles extracted from the X-ray structure of **2** reported in literature [22].

Table S4. H(X)-C-C-H(Y) dihedral angles deduced from the vicinal $^{3}J_{H-H}$ coupling constants of **3**

X-Y	NMR ^{a}	X-ray ^{b}	Ab initio
1α-2α	55.4	59.5	54.1
1β-2α	65.5	64.1	64.3
1β-2β	51.8	59.1	52.3
6α-7α	58.6	56.1	57.3
6α-7β	75.2	50.9	77.2
6β-7β	49.9	56.9	45.5
7α-8	161.1	170.8	171.8
7β-8	65.5	59.3	51.6
8-9	152.1	178.5	156.2
8-14	153.5	173.3	158.3
9-11α	57.9	56.0	57.8
9-11β	165.0	166.5	159.1
11α-12α	48.5	59.1	49.9
11α-12β	71.4	78.8	70.3
11β-12β	58.6	48.2	30.0
14α-15α	39.4	30.7	36.0
14α-15β	165.0	169.1	166.9
15α-16α	23.4	2.2	23.4
15α-16β	109.9	118.9	110.4
15β-16α	125.2	133.1	146.2
16α-17α	32.2	33.4	33.4
16β-17α	142.6	154.6	158.3

^{a} The dihedral angles in degree deduced from the vicinal $^{3}J_{H-H}$ coupling constants according to Bothner-By equation [20].

^{b} The dihedral angles extracted from the X-ray structure of **3** reported in literature [23].

Table S5. H(X)-C-C-H(Y) dihedral angles deduced from the vicinal $^{3}J_{H\text{-}H}$ coupling constants of **4**

X-Y	NMR [a]	X-ray [b]	Ab initio
1α-2α	60.7	56.7	64.7
1α-2β	?	176.7	-
1β-2α	65.5	61.2	61.2
1β-2β	62.3	58.9	64.7
2α-3	57.8	57.7	59.4
2β-3	157.0	177.7	178.9
3-4α	52.8	55.7	53.7
3-4β	154.2	174.5	150.4
6-7α	79.5	73.8	82.1
6-7β	50.2	44.8	50.4
7α-8	150.8	162.3	175.9
7β-8	52.5	43.7	53.1
8-9	154.2	176.7	151.2
8-14	161.1	175.0	157.0
9-11α	55.8	55.9	53.5
9-11β	165.0	173.5	164.0
11α-12α	60.4	54.4	60.4
11α-12β	67.8	64.9	69.6
11β-12α	?	172.0	?
11β-12β	60.0	52.6	38.3
14α-15α	39.4	39.7	35.2
14α-15β	152.8	162.4	159.5
15α-16α	23.0	20.7	20.0
15α-16β	107.5	141.3	107.8
15β-16α	124.9	129.8	108.4
15β-16β	?	8.6	?
16α-17α	27.0	24.8	27.8
16β-17α	144.3	146.0	137.9

[a] The dihedral angles in degree deduced from the vicinal $^{3}J_{H\text{-}H}$ coupling constants according to

Bothner-By equation [20].
[b] The dihedral angles extracted from the X-ray structure of 3β-hydroxygregn-5-en-20-one reported in literature [26].

Table S6. H(X)-C-C-H(Y) dihedral angles deduced from the vicinal $^3J_{H\text{-}H}$ coupling constants of **5**

X-Y	NMR [a]	X-ray [b]	Ab initio
1α-2α	18.0	0.0	18.8
6α-7α	55.0	51.7	54.1
6β-7β	49.3	52.8	49.2
7α-8	165.3	172.0	165.7
7β-8	59.3	54.7	49.2
8-9	153.5	177.6	155.9
8-14	154.9	174.7	159.2
9-11α	63.1	49.6	65.1
11α-12α	72.2	72.5	72.5
11α-12β	62.1	44.7	60.9
14α-15α	39.4	38.0	36.4
14α-15β	159.4	159.1	159.3
15α-16α	27.4	4.2	15.5
15α-16β	103.7	115.9	106.7
15β-16α	124.9	125.2	146.9

[a] The dihedral angles in degree deduced from the vicinal $^3J_{H\text{-}H}$ coupling constants according to Bothner-By equation [20].

[b] The dihedral angles extracted from the X-ray structure of **5** reported in literature [24].

Table S7. H(X)-C-C-H(Y) dihedral angles deduced from the vicinal $^{3}J_{H\text{-}H}$ coupling constants of **7**

X-Y	NMR ^{a}	X-ray ^{b}	Ab initio
1α-2α	55.4	54.7	55.2
1α-2β	?	176.8	173.2
1β-2α	65.5	69.8	65.7
1β-2β	51.8	54.5	52.2
6α-7α	55.0	52.1	55.3
6α-7β	?	74.0	63.6
6β-7β	49.3	58.9	52.8
7α-8	165.3	178.9	164.0
7β-8	59.3	51.0	55.8
8-9	153.5	173.2	152.6
8-14	154.9	177.7	154.4
9-11α	63.1	54.1	63.6
11α-12α	72.2	49.1	73.4
11α-12β	62.1	61.3	58.3
14α-15α	39.4	35.9	43.6
14α-15β	159.4	166.5	158.9
15α-16α	27.4	4.3	27.6
15α-16β	103.7	119.7	107.3
15β-16α	124.9	130.6	148.1
15β-16β	?	6.6	13.0

^{a} The dihedral angles in degree deduced from the vicinal $^{3}J_{H\text{-}H}$ coupling constants according to Bothner-By equation [20].

^{b} The dihedral angles extracted from the X-ray structure of **7** reported in literature [25].

Table S8. $^1H/^1H$ NOE constraints deduced from 2D NOESY spectra of steroidal compounds

Atom	NOE constraints of compound **1**
H1β	H11α, H19
H2β	H19
H4α	H6α, H6β
H4β	H6α, H6β, H19
H6α	H4α, H4β, H19
H6β	H4α, H4β, H19
H7β	H15β
H11α	H1β, H19
H14	H12α, H18
H15β	H7β, H18
H17	H12α
H18	H8, H11β, H12α, H14, H15β, H16α
H19	H1β, H2β, H4β, H6α, H6β, H11α, H11β, H12β

Atom	NOE constraints of compound **2**
H3α	H6α, H6β, H19
H5α	H9, H19
H6α	H3α
H6β	H3α
H7α	H15α
H7β	H15α
H7β	H15β
H8	H1β, H19
H11α	H1α
H9	H1α, H5α
H15α	H7α, H7β
H16β	H18
H18	H8
H19	H5α, H8

Atom	NOE constraints of compound **3**
H1α	H9, H19
H1β	H6β, H8, H9, H11α, H11β, H16α, H16β, H19
H2α	H9, H19
H2β	H9, H11α, H19
H6α	H14
H6β	H1β, H14, H15α, H19
H7β	H14, H11α, H15α, H15β
H11α	H1β, H15β, H18, H19
H11β	H1β, H18, H19
H12β	H15α, H15β, H16α, H16β

H15β	H7β, H11α, H12β
H16β	H1β, H7β
H17	H9, H11α, H11β, H12α, H12β, H14
H18	H8, H11α, H11β, H12β, H15α, H15β, H16α, H19
H19	H1α, H2α, H2β, H6β, H8, H9, H11α, H11β, H16α, H16β, H18

Atom	NOE constraints of compound **4**
H1β	H11α
H2α	H11α
H3	H18, H19
H4α	H19
H4β	H19
H6	H1α, H1β, H2β, H9, H11β, H12α, H12β, H15β, H16β, H19
H7α	H14
H7β	H15α, H15β
H11α	H1β, H2α
H12α	H19, H21
H14	H7α, H18, H19
H15β	H7β, H18, H19
H16β	H4β, H18
H17	H2β, H4β, H14
H18	H2β, H3, H7α, H8, H11α, H11β, H14, H15β, H19
H19	H3, H8, H12α, H14, H16β
H21	H12α, H12β, H16α, H16β, H18, H19

Atom	NOE constraints of compound **5**
H1	H11α.H19
H4	H6α
H6α	H4
H6β	H19
H7α	H14
H7β	H15α, H15β, H19
H8	H18
H11α	H1
H12α	H19, H21a
H12β	H14
H14	H12β, H19
H15α	H7β, H19
H15β	H7β, H18
H16α	H18
H16β	H18
H18	H8, H15β, H16α, H16β
H19	H1, H6β, H7β, H12α, H14, H15α
H21a	H12α

Atom	NOE constraints of compound **7**
H1α	H9
H1β	H19
H2α	H9
H2β	H19
H4	H6α
H6β	H19
H7α	H14
H7β	H15α
H8	H15α, H15β, H18, H19
H9	H1α, H2α, H14, H15α
H11α	H1α, H1β, H19
H14	H7α, H7β, H9
H15α	H7β, H8, H9
H15β	H8, H18
H16α	H19
H16β	H18, H19
H18	H21a
H19	H1β, H2β, H6β, H8, H16α, H16β
H21a	H12α, H18
